# Psychological Distress in Patients with Symptomatic Vitreous Floaters

**DOI:** 10.1155/2017/3191576

**Published:** 2017-12-10

**Authors:** Yong-Kyu Kim, Su Young Moon, Kyung Mi Yim, Su Jeong Seong, Jae Yeon Hwang, Sung Pyo Park

**Affiliations:** ^1^Department of Ophthalmology, Hallym University College of Medicine, Kangdong Sacred Heart Hospital, No. 150 Seongan-ro, Gangdong-gu, Seoul 134-701, Republic of Korea; ^2^Department of Psychiatry, Hallym University College of Medicine, Kangdong Sacred Heart Hospital, No. 150 Seongan-ro, Gangdong-gu, Seoul 134-701, Republic of Korea

## Abstract

**Purpose:**

To evaluate the degree of psychological distress in symptomatic vitreous floater patients and to evaluate whether these psychological factors are associated with the severity of discomfort associated with vitreous floaters.

**Methods:**

We recruited 61 patients with symptomatic vitreous floaters and 34 controls. The degree of posterior vitreous detachment (PVD) was evaluated using optical coherence tomography. We measured the level of depression, perceived stress, state, and trait anxiety and the degree of floater-associated discomfort with self-administered questionnaire. We compared psychological parameters between floater patients and control. We also compared clinical and psychological characteristics among different floater-associated discomfort severity groups.

**Results:**

Symptomatic vitreous floater patients showed higher rate of complete PVD and higher psychological distress compared to the control. On multiple logistic regression analysis, complete PVD (*p* = 0.001), depression (*p* = 0.001), and younger age (*p* = 0.037) were significantly associated with symptomatic floaters. There were no significant differences in complete PVD rate among different discomfort groups, while severe discomfort group showed higher depression, perceived stress, and state and trait anxiety compared to the other two milder symptom groups.

**Conclusions:**

Symptomatic vitreous floater patients showed substantial level of psychological distress, and the severity of floater symptoms was significantly associated with psychological distress.

## 1. Introduction

Vitreous floaters are visual phenomena caused by degenerative changes of the vitreous gel. Over time, vitreous collagen type IX decreases, resulting in the surface exposure of “sticky” type II collagen. These result in the vitreous collagen fibrils to aggregate and liquefy, and accompanied vitreous shrinkage and clumping make tiny shadows on the retina [1, 2]. Posterior vitreous detachment (PVD) entails the collapse of the vitreous body and anterior displacement of the posterior vitreous cortex and is the most common cause of vitreous floaters [1]. Myopic vitreopathy and asteroid hyalosis are also common causes of vitreous floaters [3]. Vitreous floater symptoms usually subside with passage of time; however, some patients suffer from persisting discomfort and seek medical care.

Although symptomatic vitreous floaters might be associated with retinal breaks or serious retinal detachment, especially when floaters increase suddenly and possibly accompanied by light flashes [4], it is usually thought to be harmless and natural aging process, and clinicians usually pay little attention to patients' discomfort. However, subjective discomfort of floater patients is considerable. In a recent study, PVD was associated with significant reduction in contrast sensitivity function [5, 6]. In addition, functional quality of life of floaters assessed by utility values was comparable to age-related macular degeneration and several systemic diseases, suggesting significant negative impact of floaters on quality of life of patients [7, 8].

We often encounter floater patients whose levels of distress are out of proportion with the severity of the floaters and very nervous for their floater symptoms. In one explorative study that investigated floater symptom from a psychological perspective, authors found that individual's ways of experiencing and reacting to floaters might be different according to the ways of how they react and respond to the disease [9]. There are possibilities that psychological factors might be associated with floater symptoms; however, psychological analyses on symptomatic vitreous floater patients are scarce. We hypothesized that psychological distress might be higher in symptomatic vitreous floater patients compared to the controls, and there will be a good correlation between the degree of floater symptoms and psychological problems. Thus, we assessed symptomatic vitreous floater patients for psychological symptoms such as depression, anxiety, and perceived stress and evaluated whether these psychological problems are related with the severity of discomfort associated with vitreous floaters.

## 2. Methods

### 2.1. Participants

Between December 1, 2015, and June 30, 2016, patients who visited our clinic for their vitreous floater symptoms and who agreed to participate in the study were consecutively enrolled. Healthy volunteers without symptomatic vitreous floaters were also enrolled as controls. The study was approved by the institutional review board (IRB) of Kandong Sacred Heart Hospital (Seoul, South Korea, IRB number 2015-12-008). All study conducted adhered to the tenets of the Declaration of Helsinki, and written informed consent was obtained from all study participants.

### 2.2. Inclusion and Exclusion Criteria

The study group consisted of patients with symptomatic vitreous floaters who primarily visited Kangdong Sacred Heart Hospital Retina Clinic to evaluate their floater symptoms and who agreed to participate in the study. In this study, we only included those with endogenous causes of vitreous floaters [1]. Patients with exogenous causes of vitreous floaters, such as vitreous or preretinal hemorrhages, inflammatory cells were excluded from the study. Those with any other ophthalmologic disorders that might affect vision and vitreous structures (e.g., diabetic retinopathy, retinal vascular obstructions, epiretinal membrane, and vitreomacular traction syndrome) and those who underwent intraocular surgeries except uncomplicated cataract surgery were also excluded from the study. The controls were collected from healthy volunteers which are those who visited the ophthalmology clinic for medical check-up and lacked eye diseases and vitreous floater symptoms and agreed to participate in the study.

### 2.3. Vitreous Floaters Symptom Questionnaire

**Figure 1:**
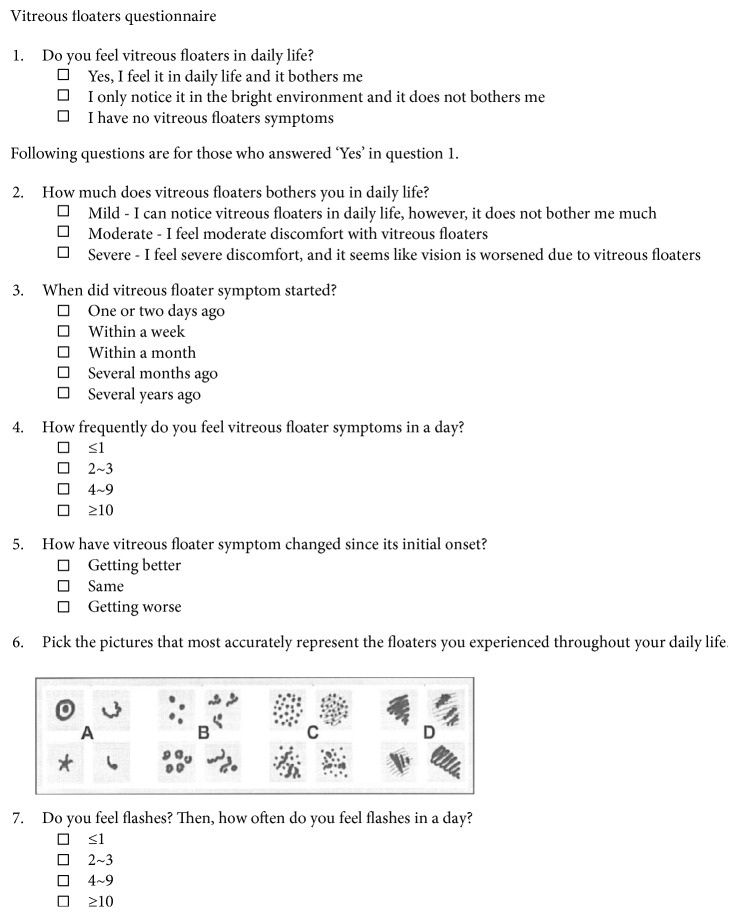
Vitreous Floaters Symptom Questionnaire. This is the vitreous floaters questionnaire used in this study, which was translated from Korean to English. The drawing in question 6 was adopted from van Overdam et al. [4].

We developed the Vitreous Floaters Symptom Questionnaire to evaluate symptoms associated with vitreous floaters. In this study, we defined symptomatic vitreous floaters as vitreous floaters that are noticed in daily life and make visual discomfort. In contrast, vitreous floaters that are noticed only in a bright environment and do not make visual discomfort were considered nonsymptomatic and excluded from the study group. Those with no floaters or nonsymptomatic floaters consisted the control group. For those with symptomatic vitreous floaters, we further investigated the degree of associated discomfort, onset, frequency, changes, and number and characteristics of vitreous floaters and frequency of associated photopsia. The degree of perceived discomfort was evaluated into three grades: mild, moderate, and severe. When patients noticed vitreous floaters in daily life, however, accompanied discomfort is minimal, it was graded mild. If vitreous floater-associated disturbance is severe enough that patients feel that their vision is getting worse, it was graded severe. We adopted a figure depicting serial groups of vitreous floaters from previous literature [4] and investigated number and shapes of vitreous floaters that patient perceived (Figure 1).

### 2.4. Ophthalmologic Examinations and Posterior Vitreous Detachment Evaluation

**Figure 2:**
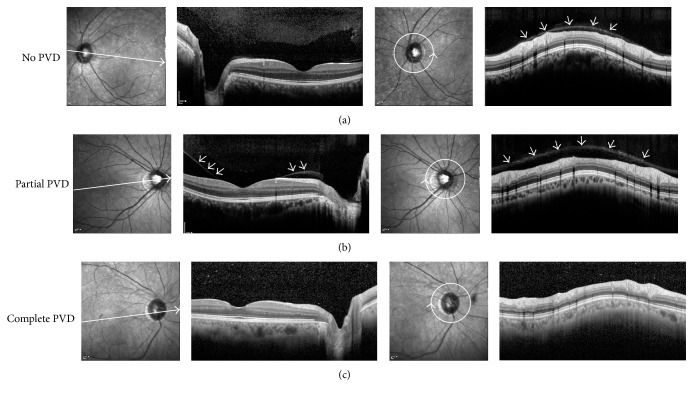
Posterior vitreous detachment grading by optical coherence tomography. Posterior vitreous status was graded with optical coherence tomography. (a) No posterior vitreous detachment (PVD), with complete attachment of the posterior vitreous cortex (PVC) to the perifoveal area, fovea, and optic disc. Circumferential peripapillary scan also confirms attached PVC (arrows). (b) Partial PVD with separation of the PVC over perifoveal area (arrows). Circumferential peripapillary scan shows attached PVC (arrows). (c) Complete PVD with detachment of PVC over perifoveal area, fovea, and optic disc, showing optically empty space above. Circumferential peripapillary scan also lacks attached overlying PVC.

Patients underwent examination by slit lamp, fundoscopy, and OCT. Patients underwent careful fundus examination to find out any evidence of PVD (e.g., Weiss ring) or any evidence of peripheral retinal breaks. Best-corrected visual acuities were measured with Snellen chart and converted to logarithm of minimal angle resolution for statistical evaluation. They were examined for any peripheral retinal tears or degeneration. The degree of PVD was evaluated using OCT (Spectralis OCT, Heidelberg Engineering, Heidelberg, Germany) with enhanced vitreous imaging technique according to the previous literature [10]. The OCT examination has an advantage on detecting a shallow PVD that is undetectable by slit lamp biomicroscopy [10, 11]. In brief, we obtained a single 30° line scan taken with a 7° tilt, traversing both the optic disc and fovea simultaneously. The image was defocused to focus on the vitreous. In addition, we also obtained circumferential peripapillary scan to ensure vitreous status at peripapillary region. We divided PVD state into three categories: no, partial, and complete PVD. No PVD was defined as complete attachment of the posterior vitreous cortex to the perifoveal area and optic disc. Partial PVD was defined as separation of posterior vitreous cortex from perifoveal/foveal area but attachment at optic disc. Complete PVD was defined as complete detachment of posterior vitreous cortex from perifovea, fovea, and optic disc (Figure 2). The PVD assessment was made by one observer (Y-K.K.) with masked patient information.

### 2.5. Psychological Evaluation

We evaluated depressive symptoms using the validated Korean version of the Patient Health Questionnaire-9 (PHQ-9), which consists of brief, self-administered 9 items that are directly from the Diagnostic and Statistical Manual of Mental Disorders, 4th Edition (DSM-IV), describing the signs and symptoms of major depression. Each item in PHQ-9 scores ranging from 0 to 3, and PHQ-9 scores of 5, 10, 15, and 20 represent mild, moderate, moderately severe, and severe depression, respectively. PHQ-9 is a reliable and valid measure of depression severity [12, 13]. We evaluated perceived stress levels using the Perceived Stress Scale (PSS), which was originally developed by Cohen et al. [14]. It measures perceived stress levels in the past month. It was originally made up of 14 items; however, shortened versions of the PSS were subsequently developed. In this study, we used the Korean version of the PSS-10 [15], which consists of 10 items and the items are rated on a five-point Likert-type scale, ranging from 0 to 4, which measures the degree of perceived stress with good reliability and validity. The scores for the 10 items are summed to obtain the total score of the PSS, with a higher score indicating higher perceived stress. We evaluated state and trait anxiety using the State-Trait Anxiety Inventory (STAI) [16]. The STAI has 20 items for assessing trait anxiety and 20 for state anxiety; each item is rated on a 4-point scale. State anxiety refers to temporary anxiety experienced by a subject around the time of measurement. On the other hand, trait anxiety refers to a generalized propensity to be anxious which is an enduring condition and considered a component of one's personality. Higher scores indicate greater anxiety levels [17]. In this study, we used the Korean version of the STAI which has good reliability and validity [18]. The Vitreous Floaters Symptom Questionnaire and psychological evaluation were done before ophthalmologic examinations.

### 2.6. Statistical Analyses

We compared OCT-based PVD status, psychological parameters, and other clinical factors between patients with symptomatic vitreous floaters and controls. Student's *t*-test was used for continuous variables, and chi-square test or Fisher's exact test was used for categorical variables. We also performed multiple logistic regression analysis to investigate factors associated with symptomatic vitreous floaters. We performed similar analysis, both univariate and multivariate analysis, for investigating factors associated with complete PVD, which is identified by OCT. We compared clinical characteristics of vitreous floater symptom, such as onset, frequency, changes, accompanied photopsia, and PVD status, psychological parameters among three groups divided by the degree of floater-associated discomfort using one-way analysis of variance for continuous variables and trend analysis using Cuzick's Wilcoxon-type nonparametric trend statistic [19] for categorical variables. Statistical analyses were performed using Stata version 13.0 statistical software (Stata Corp, College Station, TX, USA). Statistical significance was defined as *p* < 0.05, and borderline significance was defined as *p* ≥ 0.05 and *p* < 0.08.

## 3. Results

In this study, we enrolled 61 patients with symptomatic vitreous floaters and 34 controls without symptomatic vitreous floaters. Demographics, clinical characteristics, posterior vitreous status, and psychological parameters of patients and controls are summarized in Table 1. There were no significant differences in terms of age, sex, underlying diabetes or hypertension, total years of education, proportion of outdoor occupation, and visual acuity between two groups. However, patients with symptomatic floaters showed a higher rate of complete PVD identified by OCT (79% in the floater group versus 47% in the controls, *p* = 0.011). The symptomatic floater group also showed higher depression (6.5 ± 6.3 versus 2.3 ± 2.3, *p* < 0.001), higher perceived stress level (16.8 ± 6.7 versus 14.2 ± 4.4, *p* = 0.027), higher state anxiety (43.2 ± 12.6 versus 37.9 ± 8.0, *p* = 0.014), and borderline high trait anxiety (43.2 ± 10.8 versus 39.1 ± 8.4, *p* = 0.058) compared to the control group.

On multiple logistic regression analysis, complete PVD (odds ratio 9.83, 95% confidence interval 2.63–36.77, *p* = 0.001), depression (PHQ-9 score, odds ratio 1.32, 95% confidence interval 1.11–1.56, *p* = 0.001), and younger age (years, odds ratio 0.94, 95% confidence interval 0.89–0.99, *p* = 0.037) were significantly associated with symptomatic floaters (Table 2).

We also divided study participants according to the status of posterior vitreous assessed by OCT (no or partial PVD versus complete PVD) and compared clinical characteristics between two groups. The complete PVD group were older (59.7 ± 6.5 years versus 49.7 ± 12.8 years, *p* = 0.001) and more female predominant (female, 73% versus 52%, *p* = 0.035) compared to the no or partial PVD groups. Underlying hypertension was more prevalent in the complete PVD group. Visual acuity was also worse in the complete PVD group (logMAR, 0.07 ± 0.13 versus 0.02 ± 0.05, *p* = 0.029). However, there were no significant differences in terms of psychological parameters (Table 3). On multiple logistic regression analysis, only older age (years, odds ratio 1.1, 95% confidence interval 1.1–1.2, *p* < 0.001) was significantly associated with complete PVD identified by OCT.

We divided symptomatic vitreous floater patients according to the degree of discomfort that patients perceived. Twenty patients answered their floater symptoms as “mild,” 24 reported as “moderate,” and 17 reported as “severe.” We compared characteristics of vitreous floater symptoms according to the degree of perceived discomfort. There was a trend that patients with greater discomfort experienced more recently developed discomfort, which is more frequently observed in a day. Patients with more severe discomfort also encountered a large number of floaters that are in a grouped or cloud pattern and were also frequently associated with photopsia. There was also a trend that the more severe discomfort group felt that their floater symptom is getting worse since its initial onset (Table 4).

We compared clinical and psychological characteristics among different vitreous floater discomfort groups. There were no significant differences in terms of age, sex, underlying diabetes or hypertension, total years of education, and visual acuity. The proportion of complete PVD was higher in the severe discomfort group compared to the other groups; however, it did not reach statistical significance (*p* = 0.091). In contrast, psychological parameters were significantly different among the groups. The severe discomfort group showed higher depression, perceived stress, and state and trait anxiety compared to the other two milder symptom groups (Table 5).

## 4. Discussion

In this cross-sectional study, we evaluated the psychological parameters along with various clinical factors including posterior vitreous status assessed by OCT in symptomatic vitreous floater patients and controls. We found that symptomatic vitreous floater patients suffered more psychological problems, such as depression, stress, and anxiety compared to control group, and the degree of floater-related discomfort was well correlated with the severity of their psychological distress. To our knowledge, this was the first study that evaluated both objective anatomical status and subjective psychological aspects of symptomatic vitreous floater patients and thus we could more validly assess psychological problems of floater patients adjusting for their eye status.

The presence of complete PVD was associated with the presence of vitreous floater symptoms. The PVD is known to be the most common cause of vitreous floaters [20]. Age, female gender, and myopia have been thought to be the risk factors for PVD, which was assessed by 90-diopter biomicroscopy or B-scan ultrasonography [21–23]. Recently, studies on OCT-based PVD evaluation have been introduced [10, 11, 24]. Although OCT-based technique enables us to sensitively detect serial evolution of PVD [11], the empty space of posterior precortical vitreous pocket can be misdiagnosed as complete PVD with OCT scans [24]. In this study, we also assessed circumferential peripapillary scan to ensure vitreous status at peripapillary region. The peripapillary region is where the vitreous is attached until the vitreous is completely separated. When posterior vitreous status was confusing whether vitreous is completely detached or attached, it could be distinguished by observing the peripapillary region. Although this method needs to be verified in comparison to ultrasonographic findings, we believe that OCT could also effectively detect PVD status.

Patients suffering symptomatic vitreous floater showed a higher degree of psychological problems compared to the control group. After adjusting for various clinical factors, depression level was significantly higher in the floater group. The average PHQ-9 score of symptomatic floater patients was 6.5, which belongs in the range of subthreshold depression [12]. The PHQ-9 score was significantly higher in the severe discomfort group with an average of 10.9, which suggests a spectrum of depression patients [12]. There are possibilities that people with greater depression will rate their floater symptoms as more severe.

The presence of complete PVD was highly associated with symptomatic vitreous floaters. However, an association between complete PVD and the degree of floater-related discomfort was less significant. Complete PVD might be an important initiating factor for vitreous floater symptoms; however, the degree of vitreous condensation and debris might be different from patient to patient. Recently, ultrasound-based quantitative vitreous opacity assessment showed good correlation with contrast sensitivity and quality of life, and such technique might be helpful in further studies assessing floater-associated discomfort and vitreous opacities [25].

Unlike PVD status, psychological parameters showed good correlation with the severity of floater-associated discomfort. The severe discomfort group revealed worse psychological status compared to mild or moderate discomfort group. The severe discomfort group showed more depressive symptoms and higher perceived stress level than mild or moderate discomfort group. The severe discomfort group also revealed higher trait anxiety as well as state anxiety compared to the other groups. Then, how could we interpret these findings? Higher psychological problems in symptomatic vitreous floater patients in this study might stem from the presence of annoying floater symptom itself. However, because of the cross-sectional study design of the current study, the exact cause and effect relationship is inconclusive. However, there is still a chance that personal psychological problems also deteriorated floater symptoms. In a recent study on perception of subclinical floaters in an asymptomatic cohort, 84.1% of patients reported transparent floaters using vitreoscope method. Although the information on PVD status or the degree of vitreous opacity is lacking in that study, the result suggests high prevalence of floaters in the population, while most of them are unaware of it [26].

Cipolletta et al. approached floater symptom from a psychological perspective, and they suggested that the reason for some of the patients not resolving their problems after consulting an ophthalmologist is mainly because floater symptoms are not necessarily associated with pathology of the eye, but they are associated with the ways of experiencing and reacting to the disease [9]. In our study, the degree of perceived stress level showed good correlation with the degree of floater-associated discomfort. The PSS was designed to measure the degree to which individuals appraise situations in their lives as stressful and evaluate the degree to which individuals believe their life has been unpredictable, uncontrollable, and overloaded during the past month [14, 27]. Thus, one possible explanation is that patients suffering more severe discomfort from floaters assessed their life more stressful and unmanageable. However, this explanation did not coincide well with higher trait anxiety reported by the severe discomfort group because trait anxiety reflects their personality traits rather than current anxiety symptoms. Although symptomatic vitreous floaters could be a factor that influences on perceived stress of the group, the stress may stem from personality trait of the group that could make them more anxious about burdensome but not from critical symptoms on their eyes. Further studies on psychological aspects of floater patients whether these are aggravating or initiating factors of symptomatic vitreous floater are needed.

This study has several limitations, such as small case numbers and cross-sectional study design. We did not rule out other reasons for the mental state of these patients; thus, we were not able to show the exact cause and effect relationship between psychological problems and floater symptoms. We need to consider more detailed socioeconomic state of patients that might affect depression and anxiety levels. In addition, the Vitreous Floaters Symptom Questionnaire developed in this study needs to be validated in a number of different groups of patients to enable the effective measurement of floater symptoms regardless of the patient's psychological state. PVD evaluation using both ultrasonography and OCT might be more accurate when the posterior vitreous gel and premacular bursa are not clearly visualized in OCT.

In conclusion, the presence of symptomatic vitreous floaters was mostly determined by anatomical status, that is, the presence of complete PVD, which was also associated with aging. However, the severity of floater symptoms was more related with the degree of patients' psychological distress. Further studies whether psychological distress is an aggravating or initiating factors of symptomatic vitreous floater and whether surgical treatment that removes vitreous floaters could lessen patients' psychological problems are needed.

## Figures and Tables

**Table 1:** Comparison of demographics, posterior vitreous status, and psychologic parameters between patients with symptomatic floaters and control.

	Symptomatic floaters (*n* = 61)	No floaters (*n* = 34)	*p* values^a^
Age, yrs	56.5 ± 10.3	56.3 ± 10.0	0.951
Female, *n* (%)	41 (67)	22 (65)	0.804
Diabetes mellitus, *n* (%)	9 (15)	6 (18)	0.711
Hypertension, *n* (%)	20 (33)	7 (21)	0.206
Education, yrs	11.5 ± 3.4	10.7 ± 2.4	0.278
Outdoor occupation, *n* (%)	6 (9.8)	0	0.090
Visual acuity (logMAR)	0.06 ± 0.13	0.03 ± 0.07	0.102
Refractive errors (SEQ), diopters	−0.9 ± 3.2	0.2 ± 2.6	0.101
Pseudophakia, *n* (%)	4 (7)	1 (3)	0.652
PVD grading, *n* (%)			0.011
No PVD	9 (15)	9 (27)	
Partial PVD	4 (7)	9 (27)	
Complete PVD	48 (79)	16 (47)	
Depression (PHQ-9)	6.5 ± 6.3	2.3 ± 2.3	<0.001
Stress (PSS)	16.8 ± 6.7	14.2 ± 4.4	0.027
Anxiety (STAI)			
State anxiety	43.2 ± 12.6	37.9 ± 8.0	0.014
Trait anxiety	43.2 ± 10.8	39.1 ± 8.4	0.058

^a^Student's *t*-test and chi-square test or Fisher's exact test used for continuous and categorical variables, respectively. logMAR: logarithm of minimal angle of resolution; PHQ-9: Patient Health Questionnaire-9; PSS: Perceived Stress Scale; PVD: posterior vitreous detachment; SEQ: spherical equivalent; STAI: State-Trait Anxiety Inventory.

**Table 2:** Multivariate analysis on factors associated with symptomatic floaters.

	Odds ratio	95% confidence interval	*p* values^a^
Complete PVD	9.83	2.63–36.77	0.001
Depression (PHQ-9 score)	1.32	1.11–1.56	0.001
Age (years)	0.94	0.89–0.99	0.037

^a^Multiple logistic regression analysis using backward elimination (*p* > 0.10) based on the probability of the likelihood ratio. PHQ-9: Patient Health Questionnaire-9; PVD: posterior vitreous detachment.

**Table 3:** Comparison of demographics and psychologic parameters between patients with and without complete posterior vitreous detachment identified on optical coherence tomography.

	No or partial PVD (*n* = 31)	Complete PVD (*n* = 64)	*p* values^a^
Age, yrs	49.7 ± 12.8	59.7 ± 6.5	0.001
Female, *n* (%)	16 (52)	47 (73)	0.035
Diabetes mellitus, *n* (%)	3 (10)	12 (19)	0.371
Hypertension, *n* (%)	3 (10)	24 (38)	0.005
Education, yrs	12.3 ± 3.5	10.9 ± 2.9	0.054
Outdoor occupation, *n* (%)	2 (7)	4 (6)	>0.999
Visual acuity (logMAR)	0.02 ± 0.05	0.07 ± 0.13	0.029
Refractive errors (SEQ), diopters	−1.2 ± 2.8	−0.1 ± 3.1	0.108
Pseudophakia, *n* (%)	0	5 (8)	0.169
Depression (PHQ-9)	4.6 ± 5.2	5.2 ± 5.8	0.587
Stress (PSS)	15.2 ± 4.9	16.1 ± 6.5	0.463
Anxiety (STAI)			
State anxiety	40.1 ± 10.2	41.9 ± 12.0	0.475
Trait anxiety	40.3 ± 9.8	42.5 ± 10.3	0.319

^a^Student's *t*-test and chi-square test or Fisher's exact test used for continuous and categorical variables, respectively. logMAR: logarithm of minimal angle of resolution; PHQ-9: Patient Health Questionnaire-9; PSS: Perceived Stress Scale; PVD: posterior vitreous detachment; SEQ: spherical equivalent; STAI: State-Trait Anxiety Inventory.

**Table 4:** Comparison of characteristics of vitreous floater symptoms according to the degree of discomfort.

	Mild discomfort (*n* = 20)	Moderate discomfort (*n* = 24)	Severe discomfort (*n* = 17)	*p* values^a^
Onset of discomfort, *n* (%)				0.046
One or two days ago	3 (15)	0	6 (35)	
Within weeks	1 (5)	12 (50)	4 (24)	
Within months	2 (10)	6 (25)	0	
Several months ago	9 (45)	4 (17)	3 (18)	
Several years ago	5 (25)	2 (8)	4 (24)	
Daily frequency of discomfort, *n* (%)				<0.001
≤1	7 (35)	0	2 (12)	
2~3	8 (40)	6 (25)	0	
4~9	1 (5)	9 (38)	4 (24)	
≥10	4 (20)	9 (38)	11 (65)	
Discomfort severity change, *n* (%)				0.017
Getting better	9 (45)	2 (8)	2 (12)	
Stationary	9 (45)	13 (54)	10 (59)	
Getting worse	2 (10)	9 (38)	5 (29)	
Floater type, *n* (%)				0.006
1–3 floaters	11 (55)	7 (29)	4 (24)	
3–10 floaters	9 (45)	10 (42)	7 (41)	
>10 floaters	0	5 (21)	2 (12)	
A curtain or cloud	0	2 (8)	4 (24)	
Daily frequency of photopsia, *n* (%)				0.002
≤1	19 (95)	22 (92)	9 (53)	
2~3	0	2 (8)	5 (29)	
4~9	0	0	2 (12)	
≥10	1 (5)	0	1 (6)	

^a^Cuzick's Wilcoxon-type nonparametric trend test.

**Table 5:** Comparison of demographics, posterior vitreous status, and psychologic parameters among different discomfort severity groups.

	Mild discomfort (*n* = 20)	Moderate discomfort (*n* = 24)	Severe discomfort (*n* = 17)	*p* values^a^	Post hoc analysis^b^
Age, yrs	54.6 ± 10.1	56.4 ± 12.2	58.8 ± 10.3	0.459	
Female, *n* (%)	12 (60)	15 (63)	14 (82)	0.289	
Diabetes mellitus, *n* (%)	3 (15)	3 (13)	3 (18)	0.900	
Hypertension, *n* (%)	6 (30)	7 (29)	7 (41)	0.685	
Education, yrs	12.2 3.3	11.7 3.7	10.5 2.9	0.330	
Outdoor occupation, *n* (%)	3 (15)	2 (8)	1 (6)	0.618	
Visual acuity (logMAR)	0.07 ± 0.20	0.06 ± 0.09	0.06 ± 0.07	0.965	
Refractive errors (SEQ), diopters	−1.5 ± 3.4	−1.2 ± 3.4	0.05 ± 2.7	0.333	
Pseudophakia, *n* (%)	2 (10)	1 (4)	1 (6)	0.820	
PVD grading, *n* (%)				0.091^c^	
No PVD	4 (20)	4 (17)	1 (6)		
Partial PVD	2 (10)	2 (8)	0		
Complete PVD	14 (70)	18 (75)	16 (94)		
Depression (PHQ-9)	3.1 ± 3.5	6.3 ± 5.9	10.9 ± 7.0	<0.001	Mild, moderate < severe
Stress (PSS)	12.8 ± 5.0	16.3 ± 5.0	22.4 ± 7.1	<0.001	Mild < moderate < severe
Anxiety (STAI)					
State anxiety	37.3 ± 8.6	41.7 ± 11.4	52.4 ± 13.5	<0.001	Mild, moderate < severe
Trait anxiety	38.0 ± 7.4	41.8 ± 10.5	51.4 ± 10.2	<0.001	Mild, moderate < severe

^a^One-way ANOVA and chi-square test or Fisher's exact test used for continuous and categorical variables, respectively; ^b^post hoc analysis using LSD method. Significantly different groups are represented; ^c^Cuzick's Wilcoxon-type nonparametric trend test. logMAR: logarithm of minimal angle of resolution; PHQ-9: Patient Health Questionnaire-9; PSS: Perceived Stress Scale; PVD: posterior vitreous detachment; SEQ: spherical equivalent; STAI: State-Trait Anxiety Inventory.
